# Reply to Letter to Editor: Is SARS-CoV-2 responsible for relapses of Parkinson’s disease?

**DOI:** 10.1186/s41983-021-00380-7

**Published:** 2021-09-08

**Authors:** Walaa A. Kamel, Ismail Ibrahim Ismail, Mohamed Ibrahim, Jasem Y. Al-Hashel

**Affiliations:** 1 Department of Neurology, Ibn Sina Hospital, Gamal Abdel Nasser Street, Sabah Medical Area, Safat, Kuwait; 2 Department of Neurology, Beni-Suef University, Beni-Suef, Egypt; 3 Department of Medicine, Faculty of Medicine, Health Sciences Centre, Kuwait University, Jabriya, Kuwait

**Keywords:** COVID-19, SARS-CoV-2, Parkinson’s disease, Parkinsonism

To the Editor

We read with interest the comments made by Scorza and colleagues [1] on our article regarding worsening of parkinsonian symptoms in a patient with advanced Parkinson’s disease (PD) as the sole initial presentation of COVID-19 infection [2]. Herein, we would like to respond to some points that were raised as concerns and comments. In our case, we ruled out various possible causes for clinical deterioration, as previously mentioned in our case description, along with normal initial computed tomography (CT) of the brain on presentation. We agree that cerebrospinal fluid (CSF) examination would be helpful in such cases; however, the lack of fever, signs of meningeal irritation on presentation, in addition to the overall outcome, ruled out CNS infection.

As regards to not performing dopamine transporter (DAT) scan, our patient had a typical history of PD, with good response to dopaminergic therapy, and a dopamine transporter (DAT) scan is not mandatory for either diagnosis or follow-up. The authors postulated that confusion in our patient could be related to mismanagement of his medications; however, there was no history of any change in his usual doses, even when the family noticed the unusual worsening of his symptoms on his current regimen, which did not contain dopamine agonists. Moreover, no adjustment of his L-dopa dose was needed following admission, as the patient needed ventilatory support that required a small dose of propofol to relax his muscles.

The authors suggested that respiratory distress could be related to a central etiology, which is unlikely in our case, in the context of severe COVID-19 infection. Moreover, Guillain–Barre syndrome (GBS) was not considered, as deep tendon reflexes were present, with the absence of any obvious weakness, as we recently reported such a case [3]. Although we have no evidence that the virus had entered the brain, worsening of PD symptoms is a known complication of any systemic infection without direct invasion to CSF. That was partly explained by Brugger and colleagues [4], through different mechanisms, including enhanced neurodegeneration by peripheral inflammation, downregulation of dopaminergic receptors, altered transport of dopaminergic drugs through the blood–brain barrier, altered presynaptic reuptake of L-dopa and dopamine, respectively, and impaired packaging of neurotransmitters into vesicles, among other explanations. Central dopaminergic hypoactivity associated with PD has been related to an increased risk of inflammation. Furthermore, recently it was shown that dopamine inhibits TRAF6-mediated NF-κB activation and inflammation via the D5 dopamine receptor in macrophages, and mutations in the LRRK2 gene are also found in some bacterial infections and autoimmune disorders [5, 6]. Supporting our claim, a genome-wide association study [7] reported that LRRK2 mutations, as the most common genetic cause of both familial and sporadic PD, has a role in regulating inflammatory responses systemically and in the brain, and are associated with autoimmune diseases [8].

Since no other apparent explanation of PD deterioration was determined, in addition to the improvement of the condition after stabilizing COVID-19 infection, we could suggest a probable  association between SARS-CoV-2 infection and worsening of PD symptoms in our case.

## Data Availability

The data sets supporting the conclusion of this article are included within the article.

## Abbreviations

PD: Parkinson’s disease
COVID-19: Coronavirus disease 2019
CT: Computed tomography
CSF: Cerebrospinal fluid
CNS: Central nervous system
DAT: Dopamine transporter
GBS: Guillain–Barre syndrome
SARS-CoV-2: Severe acute respiratory syndrome coronavirus 2
